# A Genome Scan and Linkage Disequilibrium Analysis among Chromosomal Races of the Australian Grasshopper *Vandiemenella viatica*

**DOI:** 10.1371/journal.pone.0047549

**Published:** 2012-10-10

**Authors:** Ben Jackson, Takeshi Kawakami, Steve Cooper, Juan Galindo, Roger Butlin

**Affiliations:** 1 Department of Animal and Plant Sciences, University of Sheffield, Sheffield, United Kingdom; 2 Department of Evolutionary Biology, Uppsala University, Uppsala, Sweden; 3 Australian Centre for Evolutionary Biology and Biodiversity, University of Adelaide, Adelaide, Australia; 4 Evolutionary Biology Unit, South Australian Museum, Adelaide, Australia; 5 Departamento de Bioquímica, Genética e Inmunología, Universidade de Vigo, Vigo, Spain; University of Arkansas, United States of America

## Abstract

In the past decade the interest surrounding the role of recombination in speciation has been re-kindled by a new generation of chromosomal speciation models that invoke the recombination-suppression properties of some types of chromosomal rearrangement. A common prediction of recombination-suppression models is that gene exchange between diverging populations will be more restricted in regions of the genome that experience low recombination. We carried out a genome scan of three chromosomal races of the grasshopper *Vandiemenella viatica* (Orthoptera: Morabinae), occurring on Kangaroo Island, South Australia, using 1517 AFLP loci, with a view to elucidating the roles that selection and chromosomal variation have played in the formation of these taxa. An analysis of molecular variance demonstrated that chromosomal race accounted for a significant proportion of the genetic variance in the total dataset, which concurred with the findings of an earlier study. Sampling across one previously-identified hybrid zone, and the identification of outlier loci between parental races allowed us to establish that, in admixed populations, outlier loci which potentially pre-date the isolation of populations of races on Kangaroo Island exhibit higher levels of linkage disequilibrium with each other than putatively neutral loci. In turn this suggests that they might reside within genomic regions of low recombination, or be closely linked with each other.

## Introduction

The process of speciation can be described as the development of restrictions on genetic recombination [1] (after [2]). Closely related species or diverging populations often show heterogeneous divergence among genomic regions. Many factors contribute to such heterogeneity, including the homogenising effect of gene flow in neutral or universally adaptive regions, whilst alleles which are deleterious in the background of the opposing taxon experience reduced introgression; demographic effects, including genetic drift and bottlenecks; selection, due to ecological factors, or on genes responsible for isolation; variable mutation rates; and heterogeneous recombination rates, which are associated with various genomic features, such as GC content, gene density, repeat content, as well as macro-genomic features including centromeres, telomeres, and some types of chromosomal rearrangement (reviewed in [3], [4]). Understanding the relative importance of each of these forces is an important goal of speciation research.

An appreciation of recombination's central role in speciation helped bring about a revival of interest in chromosomal speciation over the last decade, following several lines of theoretical and empirical evidence that implicate the recombination-suppression properties of chromosomal rearrangements in facilitating divergence and speciation [5]–[8]. Regions of low recombination, including chromosomal rearrangements, have the potential to extend linkage disequilibrium over larger portions of the genome than freely recombining genic incompatibilities acting alone [6]. This is the central premise on which recombination-suppression mechanisms of speciation operate. If regions of low recombination are implicated in speciation via such a mechanism, they should demonstrate higher levels of differentiation between diverging taxa than co-linear regions [5], [7] (but see [10], [11]). Such a pattern has been found in sunflowers (*Helianthus* spp.), fruitflies, (*Drosophila* spp.; *Rhagoletis* spp.), mosquitos (*Anopheles* spp.), common shrews (*Sorex araneus*) and house mice (*Mus musculus*), although other empirical studies have produced contrasting results, notably in the human-chimpanzee lineage [9]–[12], reviewed in [13], and simulation studies have cast doubt on the efficacy of chromosomal rearrangements in maintaining divergence in the face of gene flow [14]. In *Heliconius* butterflies there are examples of pervasive genetic associations between loci responsible for mate choice, and loci under divergent selection [15]. Supergenes,– which have effects on multiple wing pattern traits, and are implicated in ecological divergence, are found within genomic rearrangements in *Heliconius numata* [16].

A second class of chromosomal speciation models, invoking underdominance, pre-date the recombination-suppression models. The basic premise of underdominance models is that alternate arrangements which cause direct fitness reduction when heterozygous (in hybrids between populations fixed for different karyotypes, for instance) prevent introgression between those populations, and thus facilitate speciation [17]. Underdominance models fell out of favour due to a variety of problems (reviewed in [6]). As in recombination-suppression models however, underdominant rearrangements represent barriers to gene flow, and are expected to be more differentiated than co-linear parts of the genome between populations which differ in karyotype.

The genus *Vandiemenella* (*viatica* species group) of Australian morabine grasshoppers comprises two named species (*V. pichirichi* and *V. viatica*) which together consist of twelve chromosomal forms. These forms are considered to be either races within these species or distinct species awaiting formal description [18]. With only two exceptions, these twelve taxa are distributed parapatrically in a mosaic pattern within South Australia (SA), often forming narrow contact zones at the boundaries of their ranges [17]. White and colleagues carried out extensive studies of a number of the hybrid zones on Kangaroo Island (KI) and the mainland of SA, as well as controlled breeding studies of hybrids between several chromosomal races, providing background data on the chromosomal variation and fitness of hybrids. These attributes, in addition to extensive chromosomal variation, make *Vandiemenella* an ideal model system for exploring the potential role of chromosomes in speciation.

**Figure 1:**
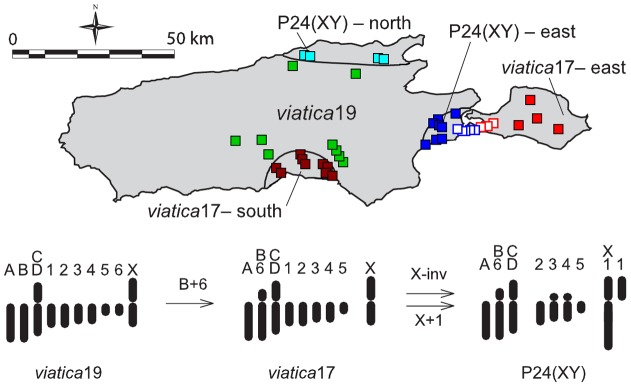
Map of the distribution of *Vandiemenella viatica* on Kangaroo Island, South Australia. Sampling sites (approximately positioned) for the five populations of three chromosomal races are colour-coded by population: *viatica*17-east (light red), *viatica*17-south (dark red), *viatica*19 (green), P24(XY)-east (dark blue), P24(XY)-north (light blue). Hybrid populations, as determined by STRUCTURE analysis, are white-filled squares. Below is the evolutionary sequence of chromosomal arrangements which gave rise to the three races, according to White *et al*. [26]. Adapted with permission from Kawakami *et al*. [19].

Three chromosomal races (P24(XY), *viatica*17, and *viatica*19) occur on KI, in five geographically distinct populations (fig. 1). There are two populations each of *viatica*17 (-east and -south) and P24(XY) (-east and -north), and one of *viatica*19. *Viatica*17 differs from *viatica*19 by one fusion. P24(XY) differs from *viatica*17 by an additional fusion and an inversion of the X chromosome (see fig. 1 for the proposed evolutionary history of these races' karyotypes). Genetic studies of these populations have suggested that a reduction in gene flow between races is associated with either chromosomal variation *per se*, or genetic variation accompanying chromosomal variation [19]. However, in the case of the contact zone between *viatica*17-east and P24(XY)-east, chromosomal and multiple nuclear markers exhibit clines of similar width and position, which suggests that restrictions on gene flow are not confined to rearranged chromosomes alone [20]. Mate-choice experiments have provided some evidence of pre-zygotic isolation in *viatica*17– *viatica*19 and *viatica*19– P24(XY) crosses, due to a failure to transfer sperm to female sperm storage organs and the production of parthenogenetic embryos. Neither of these abnormalities was detected in *viatica*17– P24(XY) crosses [21], [22].

The available evidence for the divergence of *Vandiemenella* species is compatible with secondary contact after a period of divergence in allopatry [20], [23]. This does not support White's original stasipatric model for the divergence of these taxa [17], [24] in which a sympatric origin of chromosomal races of *V. viatica* was suggested. Feder *et al*. [25] recently suggested a mechanism whereby secondary contact favours the divergence of chromosomal arrangements harbouring adaptive gene complexes under a much wider range of evolutionary scenarios than allowed by previous models. However, the extent to which the newer recombination-suppression models of chromosomal speciation are applicable to this system remains unresolved. Kawakami *et al*. [20] proposed the production and mapping of many molecular markers across the entire *viatica* genome, in order to look more closely at how differentiation between chromosomal races is related to areas of low recombination, particularly in a secondary contact scenario. We present here a genome scan of the chromosomal races of *V. viatica* on KI using 1517 AFLP loci to address this question.

We had three specific aims: 1) to characterise the population structure of *V. viatica* on KI and identify putative hybrid individuals, 2) to detect F_ST_ outlier loci (which represent putative barriers to introgression between races), and 3) conduct linkage disequilibrium (LD) analysis, specifically to test for widespread associations between outlier loci. We controlled for the expectation that outlier loci would exhibit higher than average linkage disequilibrium with each other in hybrid grasshoppers because of migrants bringing allele combinations from parental populations. We found strong population structure associated with chromosomal race, and to a lesser extent with separate populations of individual chromosomal races. We also identified both hybrid individuals and outlier loci between one population pair (*viatica*17-east – P24(XY)-east). LD analysis between outliers in these hybrids suggested that differentiated loci exhibit a higher level of association with each other than is expected due to migration alone. This implies that outlier loci are genomically clustered, in close physical linkage with each other, or reside within regions of low recombination.

## Results

### Population Structure

**Figure 2:**
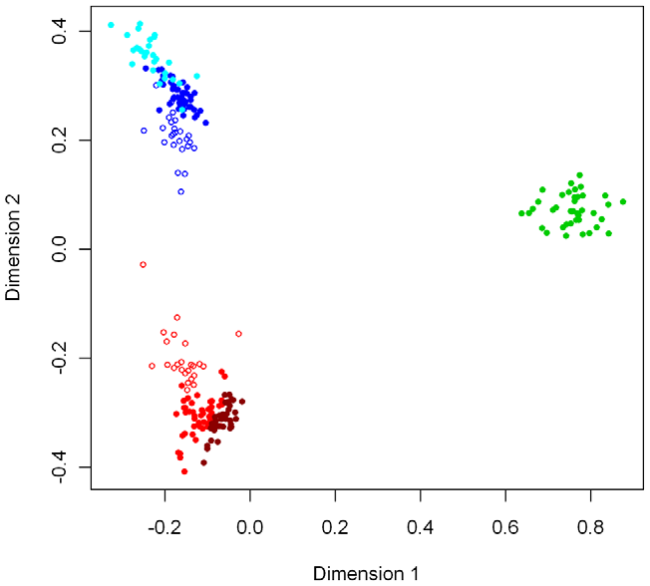
Multi-dimensional scaling (in two dimensions) of pair-wise relatedness between all individuals. Individual grasshoppers are colour-coded by population: *viatica*17-east (light red), *viatica*17-south (dark red), *viatica*19 (green), P24(XY)-east (dark blue), P24(XY)-north (light blue). Open symbols represent individuals with hybrid genotypes, defined from STRUCTURE runs. Chromosomal races and populations of chromosomal race are identifiable as distinct clusters, and hybrids between P24(XY)-east and *viatica*17-east tend to lie in between their parental population clusters.

Pair-wise relatedness between all individuals calculated using AFLP-SURV was visualised in two dimensions using multi-dimensional scaling. The resulting plot separated the three chromosomal races of *V. viatica* on KI, as well as the two geographically separate populations of *viatica*17 (-east, and -south) and P24(XY) (-east, and -north) within each of their respective race-specific clusters (fig. 2). Individuals from sampling sites closest to the contact zone between P24(XY)-east and *viatica*17-east tended to lie closest to the opposing population's cluster (fig. 2), which implied introgression.

**Figure 3:**
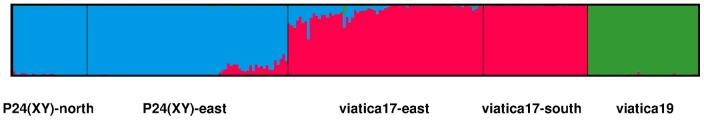
Genetic admixture proportions of individual grasshoppers. From STRUCTURE runs of all 249 genotyped individuals, from the five populations of three chromosomal races on Kangaroo Island, and with K = 3 clusters. Hybrid individuals are visible between the P24(XY)-east and *viatica*17-east populations. These individuals were collected from populations geographically closest to the hybrid zone described in Kawakami *et al*. [20] (fig. 1).

Population clustering analysis identified three clusters (K = 3) as the uppermost hierarchical level of population structure for all 249 individuals. These clusters corresponded to the three chromosomal races sampled on KI (fig. 3). This analysis also identified putative hybrid individuals between P24(XY)-east and *viatica*17-east. Using individual Q values (the admixture proportions for each individual) from a single analysis of all P24(XY)-east (n = 73) and *viatica*17-east (n = 71) individuals with K = 2 (data not shown), we assigned 51 grasshoppers as hybrids, out of 144 individuals in total sampled from the two populations Twenty-six hybrid individuals were sampled from the *viatica*17-east side of the hybrid zone, and 25 from the P24(XY)-east side. Hybrid sample sites were subsequently defined as those containing individuals that had no less than 2% of the genome assigned as belonging to either population's cluster. Hybrid sample sites were treated together as a separate population for subsequent analyses (see methods).

Pair-wise F_ST_ values between populations varied from 0.37 to 0.51 for inter-chromosomal-race comparisons, excluding the hybrid zone samples we identified from STRUCTURE analysis. F_ST_ values between the two populations each of P24(XY) and *viatica*17 were 0.12 and 0.28, respectively. Analysis of Molecular Variance (AMOVA) revealed that chromosomal race explained 37% of the total genetic variation, although the covariance component associated with this hierarchical level was not significant (p = 0.07). An additional 16% of genetic variation was explained by differences among populations within chromosomal races, and the covariance component at this level was found to be significantly greater than expected (p<0.001). Differences between sampling localities (excluding hybrid zone samples) within the five populations explained 7% of the variation, and the covariance component at this level was also significant (p<0.001). 38% percent of genetic variation was partitioned within sampling localities.

### Genomic outliers

Binary allele counts for the four pairs of populations which form contact zones on KI (fig. 1) were subjected to outlier analysis in order to identify strongly differentiated loci between the P24(XY), *viatica*17 and *viatica*19 genomes. Analyses were also conducted between all other pairs of populations of the three chromosomal races (four non-geographically-contiguous inter-race pairs and two non-geographically-contiguous intra-race pairs).

**Figure 4:**
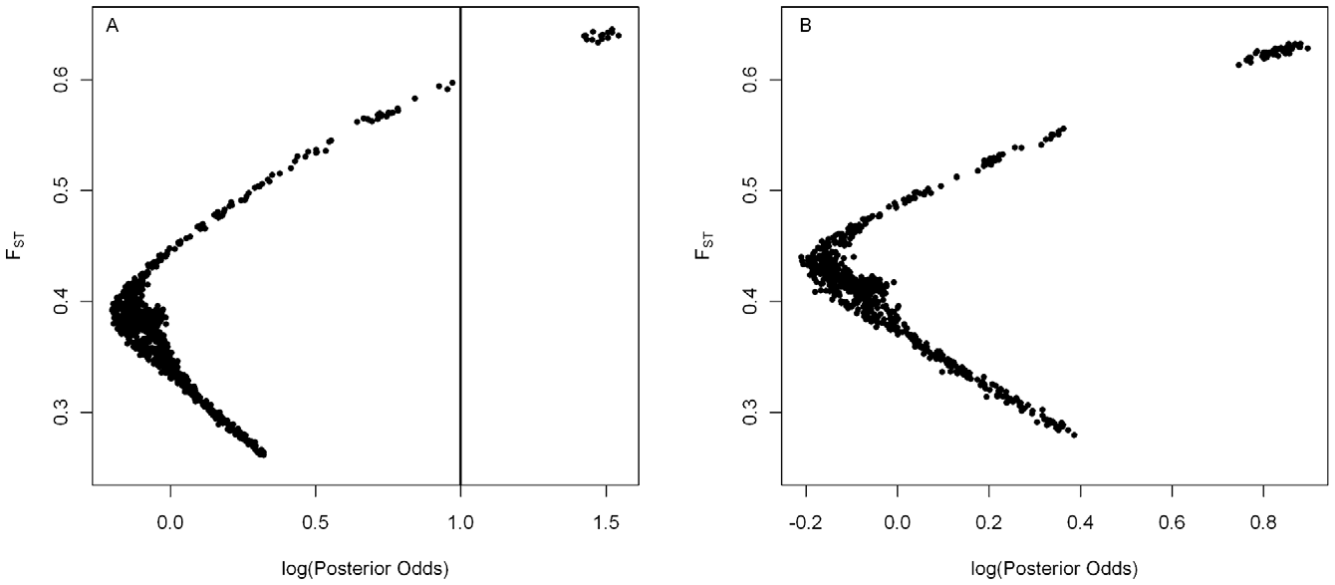
F_ST_ outlier locus identification. Locus-specific F_ST_ plotted against the posterior odds of the model including locus-specific selection effects versus the model excluding locus-specific selection effects, for A) P24(XY)-east vs. *viatica*17-east: the cluster of loci to the right of the vertical line at log(PO) > 1 are the 14 loci classified as significant outliers from this population pair, and B) P24(XY)-north vs. *viatica*17-south: the cluster in the top right corner includes seven loci which are also significantly differentiated in the P24(XY)-east vs. *viatica*17-east pair.

The comparison between *viatica*17-east and P24(XY)-east returned 14 significantly differentiated loci (log Posterior Odds > 1– see methods), which formed a tight cluster in the posterior probability – F_ST_ plot (fig. 4A). These 14 loci remained significant after applying a false discovery rate (FDR) P<0.05, and represented 1.4% of the total number of loci which were polymorphic between these populations. The comparison between P24(XY)-north and *viatica*19 returned two significantly differentiated loci (log PO > 1); however, neither was significant after applying a FDR P<0.05. These two loci were not found in the comparison between *viatica*17-east and P24(XY)-east. In the remaining two pair-wise comparisons between populations that form putative contact zones on KI, no outliers were detected.

Of the inter-race comparisons between populations which do not form putative contact zones on KI, the comparison between P24(XY)-north and *viatica*17-east returned 24 outlier loci (log PO > 1), 11 of which were common to the P24(XY)-east – *viatica*17-east outlier analysis. All 24 remained significant after applying an FDR P<0.05. The comparison between *viatica*19 and *viatica*17-east returned 34 outliers, eight of which remained significant after applying an FDR P<0.05. Comparisons between P24(XY)-east and *viatica*17-south, and P24(XY)-north and *viatica*17-south did not return any differentiated loci (log PO > 1), although in both instances distinct clusters of loci were observed at high F_ST_ and posterior probability values (fig. 4B). The two intra-race comparisons: P24(XY)-east – P24(XY)-north; and *viatica*17-east – *viatica*17-south, returned nine and 14 outlier loci respectively (log PO > 1), all of which remained significant after applying an FDR P<0.05. None of these loci were shared between the two intra-race comparisons (Table S1).

### Linkage Disequilibrium

**Figure 5:**
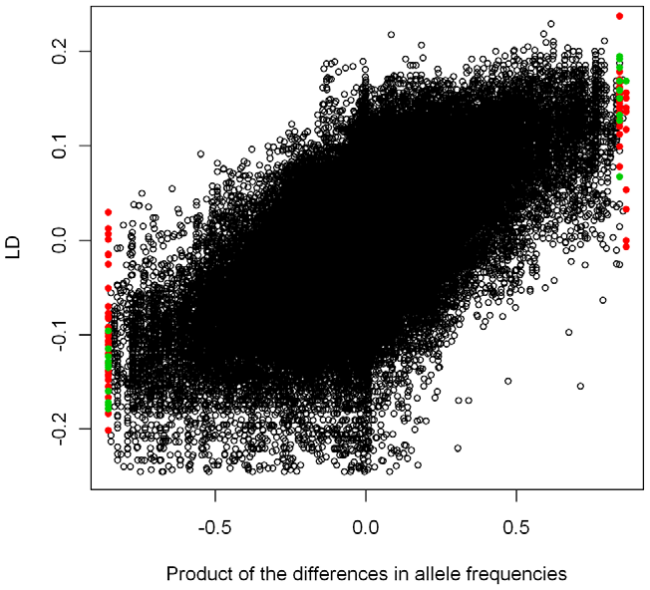
Linkage disequilibrium between outlier locus pairs versus neutral locus pairs in hybrid individuals. The product of differences in allele frequencies [(p–q)*(r–s)] between parental populations for all pair-wise combinations of loci between P24(XY)-east and *viatica*17-east, versus linkage disequilibrium (LD) between the same locus pairs in hybrid individuals. Black open circles: non-outlier locus pairs, red filled circles: pairs of outlier loci identified only in the *viatica*17-east and P24(XY)-east comparison, green filled circles: pairs of outlier loci identified both in the *viatica*17-east and P24(XY)-east comparison and the *viatica*17-south and P24(XY)-north comparison. Outlier points with positive x-axis values comprise outlier locus pairs, of which both were fixed for the presence allele in the same population. Outlier points with negative x-axis values comprise outlier locus pairs which were fixed for the presence allele in opposite populations.

The 14 loci identified as outliers between P24(XY)-east and *viatica*17-east tended to exhibit higher pair-wise linkage disequilibrium in hybrid individuals than neutral loci, as evidenced by pair-wise outlier LD extending into the tails of the overall distribution of LD (fig. 5). However, LD in a hybrid zone is expected to be greater when the loci involved have steeper clines. This is likely to be the case for outlier loci. Outliers did not exhibit higher levels of LD than neutral loci when the product of allele frequency differences across the hybrid zone [(p–q) * (r–s)] was taken into account, either in the case where the presence alleles for both loci were fixed in the same population (mean LD for outlier loci: 0.105, mean LD for neutral loci: 0.115, one-tailed two-sample t-test: t = 1.025, df  = 52.24, p = 0.845) or in the case where the presence alleles for each of the two loci were fixed in opposite populations (mean LD for outlier loci: 0.135, mean LD for neutral loci: 0.118, one-tailed two-sample t-test: t = 1.296, df  = 57.42, p = 0.1) (fig. 5).

We applied the same test to the seven loci which were significant outliers in the P24(XY)-east – *viatica*17-east comparison and also occurred in the outlying cluster of loci in the posterior probability – F_ST_ plot from the P24(XY)-north – *viatica*17-south comparison. In this case, outlier locus pairs did exhibit significantly higher LD than neutral locus pairs with an equivalent range of [(p–q) * (r–s)], both in the case where the presence alleles for both loci were fixed in the same population (mean LD for outlier loci: 0.142, mean LD for neutral loci: 0.113, one-tailed two-sample t-test: t = −3.10, df  = 10.60, p = 0.005), and in the case where the presence alleles for each of the two loci were fixed in opposite populations (mean LD for outlier loci: 0.154, mean LD for neutral loci: 0.119, one-tailed two-sample t-test: t = 3.18, df  = 10.86, p = 0.004) (fig. 5). These results remained significant when we applied more stringent cut-off values of [(p–q) * (r–s)] >0.8 and [(p–q) * (r–s)] <−0.8 for the neutral loci that we compared against outlier loci for pair-wise LD (mean LD for outlier loci: 0.142, mean LD for neutral loci: 0.112, t = −2.929, df  = 14.20, p = 0.006; mean LD for outlier loci: 0.154, mean LD for neutral loci: 0.112, t = 3.62, df  = 12.90, p = 0.002).

## Discussion

### Population Structure

The AFLP dataset presented here provided evidence of strong population structure in *V. viatica* on KI. Our analysis was able to resolve genetic clusters corresponding to chromosomal race, and geographically isolated populations of two of these races (fig. 2) consistent with similar analyses carried out using fifteen variable allozyme loci between the same populations of *V. viatica* present on KI [19].

The amount of variation which could be explained by chromosomal race according to AMOVA (∼37%) was in close agreement with a study of 11 chromosomal races of *Vandiemenella*, including mainland populations, which found 40–65% of nuclear genetic variation could be attributed to differentiation among chromosomal races [20]. Although the covariance component associated with chromosomal race was not significant, this may have been due to the lack of statistical power associated with testing the topmost levels of population structure in this hierarchy with only two degrees of freedom. The ability of ordination methods to distinguish chromosomal races suggests that population structure at the level of chromosomal race is substantial (fig. 2). STRUCTURE analysis detected three clusters at the uppermost level of population structure across all of the populations on Kangaroo Island, which corresponded to the three chromosomal races.

The pattern of clear separation of chromosomal races, and to a lesser extent of geographically isolated populations of *viatica*17 and P24(XY) within each of their respective race-specific clusters is the pattern expected on the basis of previous studies [19], [20]. The split between chromosomal races has been estimated to have occurred between one and three million years ago [19], with current evidence compatible with a period of divergence in allopatry followed by secondary contact [20], [23]. Separate populations within individual races were probably isolated some time later, with contact zones on KI possibly being established after the last glacial maximum about 18,000 years ago [20].

STRUCTURE analysis also identified putative hybrid individuals from the hybrid zone between *viatica*17-east and P24(XY)-east. Although it is possible that assignment of dual ancestry to these individuals was due to incomplete lineage sorting rather than introgression, the time to the split between these races is large, and putative hybrids were located exclusively in populations nearest the hybrid zone (fig. 1), both of which argue against incomplete lineage sorting as an explanation for our STRUCTURE results. No evidence of individuals of hybrid or backcross origin was identified from any other population pair, probably due to an inability to locate contact zones in the field (fig. 3). These contact zones, described by White [26], [27], have likely been displaced by changing land-use practices [19].

White and colleagues [21], [28] also argued, on the basis of their cytological examination using hybrids from different parental race pairs, that reproductive isolation is stronger between *viatica*19 and *viatica*17, as well as between *viatica*19 and P24(XY), than between *viatica*17 and P24(XY). In addition they predicted that widths of hybrid zones between *viatica*19– *viatica*17 and *viatica*19– P24(XY) population pairs should be narrower than between *viatica*17– P24(XY) population pairs, and wild hybrids of the former should be more rare. As well as potentially explaining our inability to detect hybrids between some population pairs, this apparent variation in the level of reproductive isolation is of interest because it suggests that different processes, or differing strengths of those processes, contribute to differential reproductive isolation between different chromosomal race pairs. Feder *et al*. [29] called for genomic analyses between closely related taxa that span the speciation continuum to further our understanding of the genomics of speciation, and *V. viatica* might be an appropriate candidate for this future work on this theme.

### Genomic Outliers

The lack of outliers between five out of ten population pairs was surprising, given the results of similar analyses [30]. One possible interpretation is that selection played little role in the divergence and isolation of these populations of *V. viatica*. There are no obvious habitat differences between these populations, in contrast to the majority of outlier analyses surveyed by Nosil *et al*. [30] in which the populations compared inhabit contrasting environments. Divergence without selection might be envisaged if isolating barriers between chromosomal races of *V. viatica* evolved by drift during a period of allopatry. An allopatric phase in the demographic history of chromosomal races of *V. viatica* was suggested by Kawakami *et al*. [23], and there is both theoretical [31] and empirical [32] evidence for the evolution of isolation factors under neutrality.

An alternative explanation is that high background F_ST_ values compromised the power of BayeScan to detect outlier loci. Foll and Gaggiotti [33] note that high mean genetic differentiation (F_ST_ > 0.25) affects the power of BayeScan to detect differentiated loci in simulated datasets. Perez-Figueroa *et al*. [34] also simulated the effect of high F_ST_ on the ability of outlier detection methods to identify loci subject to selection, and found that F_ST_ values of 0.25 or higher lead to high proportions of false negatives. Pair-wise F_ST_ values for inter-race comparisons (not including hybrid individuals) obtained from AFLP-SURV ranged from 0.37 to 0.51, with the P24(XY)-east – *viatica*17-east population pair exhibiting the second-lowest pair-wise F_ST_ value of any inter-race comparison (0.38). The latter value lends confidence to the validity of outlier loci detected between P24(XY)-east and *viatica*17-east, because the high pair-wise F_ST_ between these two populations should render tests for the presence of outlier loci more conservative. There are examples of the successful detection of AFLP outlier loci between populations with high background F_ST_ in the literature, such as, for example, in *Howea* palms (F_ST_: 0.31) [35].

Candidate loci produced by F_ST_ outlier methods are often explained as being linked to the products of local adaptation. Each individual F_ST_ outlier locus reflects divergence in only a small proportion of the genome. Therefore, some authors have argued that the numbers of loci classified as outliers by these methods (typically 2–10% of those studied [30]) suggests that genomes contain thousands of local-adaptation loci and that this seems unlikely [36]. However, this argument assumes that each outlier marks a separate target of selection, which may not be the case, especially if targets of selection lie in regions of low recombination. Also, several hypotheses other than local adaptation have also been advanced to explain the presence of F_ST_ outlier loci (reviewed in [36]), including contemporaneous and historic population processes, selective sweeps, and genetic incompatibilities not involved in habitat-specific selection (intrinsic incompatibilities).

The hybrid zone between *viatica*17-east and P24(XY)-east is likely to be a tension zone [20], and presumably involves multiple intrinsic genetic incompatibilities. Tension zones are effective at reducing gene flow between taxa, and intrinsic incompatibilities may be capable of reducing introgression to a greater extent than local adaptation loci [36], [37]) thus potentially better explaining F_ST_ outlier loci. Whether the outlier loci detected between *viatica*17-east and P24(XY)-east are the products of linkage with loci involved with local adaptation or intrinsic incompatibilities is outside the scope of this study.

Bierne *et al*. [36] discussed the possibility that environmental gradients act synergistically with intrinsic incompatibilities in determining the positions of hybrid zones. According to their model, a transition in habitat type may dictate the location of a hybrid zone, but the existence of the barrier to introgression itself is more likely to be determined by intrinsic incompatibilities. Interestingly, the contact zone between *viatica*17-east and P24(XY)-east on KI is coincident with a change in soil type [21]. Further, the association between soil profile and chromosomal race distribution is not found across other *viatica*17– P24(XY) transitions in South Australia [26], [38], which suggests that it is not necessary for the prevention of admixture between these two races.

The outliers detected from intra-race comparisons between the two populations each of P24(XY) and *viatica*17 may represent the products of processes subsequent to the geographic isolation of separate populations of the same chromosomal race. Whether these outliers result from selection due to their linkage to local adaption or intrinsic incompatibility loci remains an open question, and requires further investigation.

### Linkage Disequilibrium

The identification of hybrid individuals and outlier loci between P24(XY)-east and *viatica*17-east allowed us to compare levels of LD between outlier and neutral loci in hybrids, which are potentially descended from parents which were heterokaryotypic for the rearrangements which characterise races of *V. viatica* (fig. 1). Physically unlinked loci often exhibit linkage disequilibrium in hybrid zones because of the inflow of parental genotypes by migration [39]. Barton and Gale [39] and Barton [40] showed that the level of linkage disequilibrium between two loci in the centre of a hybrid zone should be directionally proportional to the product of the slopes of allele frequencies of the two loci across the zone and inversely proportional to the recombination rate between the loci. Therefore, physically clustered loci, or those associated with regions of low recombination should show higher LD than other loci with similar allele-frequency differences across a hybrid zone. The fourteen outlier loci that we identified between *viatica*17-east and P24(XY)-east are all fixed for opposite alleles in opposing populations (presence in *viatica*17-east and absence in P24(XY)-east, or vice versa). Consequently, these outliers are likely to exhibit high levels of LD with each other in the hybrid zone, regardless of their genomic location. In order to account for this, we compared LD against the product of the differences in allele frequencies [(p–q)*(r–s)] for neutral and outlier loci. This allows for migration when determining if outlier loci exhibited higher than expected LD in hybrids provided that cline widths do not vary (as observed by Kawakami et al. [20]). When we included all 14 fixed differences between *viatica*17-east and P24(XY)-east as outliers, our results did not suggest that these loci have higher pair-wise LD than pair-wise LD between neutral loci for a similar range of [(p–q)*(r–s)].

We carried out the same test after restricting our outlier loci to the subset of loci that met two criteria: 1. They were significantly differentiated in the *viatica*17-east – P24(XY)-south comparison, and 2. They were strongly differentiated in the *viatica*17-south – P24(XY)-north comparison, falling within the extreme cluster of loci in the posterior odds – F_ST_ plot (fig. 4B). Although these loci were not found to be significant candidates for selection between *viatica*17-south and P24(XY)-north, this might have been due to high background F_ST_ affecting the power of BayeScan to detect outliers (see above). Restricting the loci to those shared between the two independent *viatica*17– P24(XY) comparisons left seven outlier loci between *viatica*17-east and P24(XY)-east. The most parsimonious explanation for these loci being differentiated in both independent population comparisons is that the processes underlying their differentiation pre-date the isolation of populations of each race on KI. These outlier loci exhibited significantly higher pair-wise LD than neutral loci for a similar range of the product of allele frequencies across the hybrid zone between P24(XY)-east and *viatica*17-east.

Absolute levels of LD in our dataset approach maximal values in some cases (0.25). Mean LD between pairs of the seven outlier loci shared between independent *viatica*17– P24(XY) comparisons was between 0.14 and 0.15, compared to mean LD between neutral locus pairs with a similar range of the product of allele frequencies at around 0.11 (see results; fig. 5). These levels of LD are greater than those found in the *Bombina* hybrid zone studied by Barton and Gale [39], where D = 0.037 for unlinked loci with fixed differences between parental populations. It is also larger than the level of LD observed between AFLP outlier loci at the centre of a hybrid zone between ecological morphs of the marine snail *Littorina saxatilis*: D ≈ 0.04 [41]. It is important to note however, that levels of LD estimated for dominant markers are subject to some uncertainty, with allele frequencies calculated assuming Hardy-Weinberg equilibrium.

Inferences about the position of our AFLP loci must necessarily be tentative, because we have no way in this study of determining their actual genomic location. However, the results presented here show that those loci whose genetic differentiation predates the separation of populations of chromosomal races on KI exhibit higher pair-wise LD than the rest of the genome, which implies low recombination amongst these loci. There are various possible explanations for this finding, one of which is that loci map to the rearrangements that differentiate P24(XY) and *viatica*17, and within which there may be an effective suppression of recombination in heterokaryotypes. Other genomic and genetic features are also associated with regions of low recombination, including centromeres, simple repeats, and GC content [4], and association with one or more of these factors could also result in the same pattern of low recombination. It is also possible that these loci cluster tightly in the genome, and may therefore exhibit higher-than-expected LD due to physical proximity, without the need to invoke recombination suppression.

Epistatic selection might increase LD between incompatibility loci within a hybrid zone, but this effect is probably small compared with the effect of the migration of parental haplotypes. Noor et al. [5] found that incompatibilities between *Drosophila persimilis* and *D. pseudoobscura* were strongly associated with fixed inversion differences between the two species and absent outside inversions. This supports the expectation that genic incompatibilities will break down in the face of gene flow [42], and similar effects have been observed in hybrid zones [32].

It is interesting that outlier loci which are implicated in divergence between *viatica*17-east and P24(XY)-east subsequent to the split between separate populations of chromosomal race do not demonstrate the same pattern of higher-than-expected LD. This suggests either that they are less likely to exist in regions of reduced recombination, that they are less likely to cluster in the genome generally, or that they are less likely to be involved in epistatic interactions with each other.

### Conclusion

The population structure of *V. viatica* on Kangaroo Island revealed by our AFLP dataset reflected previous analyses, resolving chromosomal races and geographically isolated populations within those races. We were able to identify outlier loci between populations, as well as putative hybrid individuals, in one population pair – between P24(XY)-east and *viatica*17-east. This allowed to us to test for linkage disequilibrium between loci in potential heterokaryotypes, where we found higher-than-expected LD between outlier loci shared between independent *viatica*17– P24(XY) comparisons.

This observation is consistent with recombination-suppression models of speciation. These models are united in the prediction that loci which differentiate diverging populations should map more frequently to genomic regions of reduced recombination during the speciation process [5]–[8]. Feder and Nosil [14] cast doubt on the efficacy of chromosomal rearrangements in maintaining divergence in the face of gene flow, although under specific conditions – the complete elimination of recombination, coupled with strong selection and low migration – they might be expected to effectively harbour speciation genes between taxa, particularly for a limited period of time after a secondary contact event. Interestingly, the time since the isolation of *viatica*17-east and P24(XY)-east may fit into this range (up to 20,000 generations). It is worth noting that we also expect differentiation between speciating taxa to map to chromosomal rearrangements under underdominance models of chromosomal speciation. Although these models rely on a reduction in the fitness of hybrids which are heterozygous for underdominant arrangements, underdominance also results in rearranged regions experiencing a restriction in gene flow relative to the rest of the genome.

Other mechanisms are also capable of explaining the LD we observed between outlier loci. Centromeres and other factors, detailed above, are capable of modifying recombination. Our outlier loci may be associated with these genomic features, rather than with chromosomal rearrangements, or they may be physically clustered in the genome. Because these AFLP loci are anonymous in terms of their genomic location, it is not possible to distinguish these alternatives using the dataset presented here.

*Vandiemenella viatica* represents an excellent study system to investigate further the role that chromosomal rearrangements play in adaptive divergence and speciation. Next generation sequencing technologies offer the opportunity for genetic studies of non-model organisms, and might be productively applied in this system to investigate patterns of neutral and adaptive variation in rearranged and co-linear genomic regions, in order to further elucidate the genomic architecture of adaptation and speciation.

## Materials and Methods

### Samples

Grasshoppers were collected from 42 sampling sites on Kangaroo Island (KI), Australia during field seasons between 2002 and 2005. These 42 sample sites represent three chromosomal races of *V. viatica*: P24(XY), *viatica*17 and *viatica*19, with P24(XY) and *viatica*17 each consisting of two geographically isolated populations (fig. 1). There were no significant differences in allele frequency between years at a hybrid zone between P24(XY) and *viatica*17 on eastern KI [20]. In addition, previous nuclear gene (allozyme) analyses of the races showed there was strong genetic similarity among each of the two geographically isolated populations of *viatica*17 and P24(XY), despite their isolation for potentially thousands of generations [23]. Hence, it was deemed unlikely that temporal variation in allele frequencies over three years would influence the results of our population genetic analyses, which were carried out on pooled data from each of the field seasons. Among the 42 sites, eight sites represent a hybrid zone between P24(XY) and *viatica*17 on eastern KI. Individuals' karyotypes were determined for males using fresh testes by aceto-orcein staining described in White *et al*. [26] and Kawakami *et al*. [19]. DNA from single hind legs of 249 individual grasshoppers from the five populations of three chromosomal races [n: P24(XY)-east  = 73, P24(XY)-north  = 27, *viatica*17-east  = 71, *viatica*17-south  = 38, *viatica*19 = 40] on KI was extracted using the PUREGENE DNA Isolation Kit from GENTRA (Minneapolis, MN, USA). DNA extractions of 32 individuals were run twice to assess the repeatability of AFLP genotyping. Repeatability was scored as the percentage of mismatches between presence and absence alleles across these 32 individuals.

All necessary permits were obtained for the described field studies: in National Parks, samples were collected under permits from the Government of South Australia Department for Environment and Heritage (permit ref: K24813). Outside of National Parks, samples were collected on public land along roads or along the coast: no specific permission was required for these locations. *Vandiemenella viatica* is not an endangered or protected species.

### AFLP genotyping

DNA extraction and AFLP analysis up to the genotyping stage was performed according to Whitlock *et al*. [43], after Vos *et al*. [44] with the following modifications: two 6 bp-cutting (*Eco*RI and *Pst*I) restriction enzymes were used because of the large size of the *V. viatica* genome, and their volume at the digestion stage was doubled, to 0.2 µl. The digestion stage was run for 3 hours and 30 minutes at 37°C, followed by 10 minutes at 70°C. 2 µl of diluted ligation mixture was used as the template for pre-selective PCR (polymerase chain reaction) – and all quantities in the pre-selective PCR mastermix were doubled, except formamide, which was removed from the protocol and its volume replaced by an increased volume of water. The product of the pre-selective stage was diluted by a factor of 1∶8 with the addition of water, and 1 µl was dried in the bottom of a well-plate before the addition of the selective amplification stage reagents. For the selective stage, 2.95 µl of water was added per sample, and the quantities of *Eco*-FAM and *Eco*-NED were increased to 0.095 µl and 0.065 µl respectively. One µl of selective PCR product was added to 9 µl ABI HDI formamide, (with 6 µl ABI ROX 500 size standard mix added per 1 ml of formamide) and fingerprinted on an ABI 3730 DNA sequencer (Applied Biosystems, California, USA) with an injection time of 5 seconds, and a run-time of 1600 seconds.

Twelve primer combinations were used at the selective PCR stage, multiplexed two combinations at a time, using FAM and NED dyes. Genetic profiles were analysed using ABI Genemapper (Applied Biosystems). Bins were set manually in the 50–500 bp range, resulting in a total of 2125 bins over all 12 primer combinations. An output matrix of peak heights for all samples at all loci was generated in GeneMapper (Applied Biosystems) and exported to AFLPscore 1.4a [42] which is a software package for scoring dominant marker fingerprints, implemented in the R statistical environment [45]. A locus threshold and phenotype-calling threshold were applied in AFLPscore to provide repeatable loci, based on minimizing the mismatch error rate between samples and repeats. This process reduced the number of loci to 1517, with a mismatch error rate of 2.86%. The resulting binary matrix of AFLP genotypes was used for all subsequent analyses.

### Population Structure

AFLP-SURV [46] was used to generate a matrix of pair-wise relatedness between all individuals, after Lynch and Milligan [47]. This pair-wise relatedness matrix was subject to classical multidimensional scaling using the cmdscale() function in R, and was visualised in two dimensions in order to cluster individuals into genetic groups with no *a priori* knowledge of population structure. AFLP-SURV runs were conducted using the Bayesian method, with non-uniform prior distribution of allele frequencies, assuming Hardy-Weinberg equilibrium, and with 10,000 permutations for tests on F_ST_ and 10,000 bootstraps for genetic distances.

Analysis of Molecular Variance (AMOVA) was implemented using the amova function of the ade4 package [48] in R. We used squared Euclidean distances between AFLP haplotypes to conduct AMOVA. Because Euclidean distance is not the most appropriate measure of differences between AFLP haplotypes due to the asymmetrical probabilities of gaining or losing presence alleles, we first compared a variety of distance matrices (including 1-r (1-the relatedness metric produced by AFLP-SURV); squared Euclidean distance; and an asymmetric binary distance). Both phi-statistics values and the percentage co-variance explained were in close accordance for all different distance measures (data not shown). Multidimensional scaling in R also produced qualitatively similar results for each different distance measure. We implemented a 4-level hierarchy for AMOVA tests, defined as follows: level one – individual; level two – sampling site; level three – geographical population within chromosomal race; level four – chromosomal race. The significance of covariance components was assessed by the permutation method outlined in Excoffier *et al*. [49], where the distribution of covariance components obtained from permuting haplotypes within and between hierarchical levels 10,000 times is compared to the point-values of covariance components from the user-defined population structure.

We used STRUCTURE [50]–[52] to test the global population structure of *V. viatica* on KI; to identify population structure within each of the five geographical populations within chromosomal races; and to identify hybrid individuals. STRUCTURE implements a Bayesian model-based clustering algorithm and can be used with a variety of genetic marker types [50]–[52]. STRUCTURE runs including all five populations of three chromosomal races were carried out with K = 1–10, 100,000 burn in runs, 1,000,000 MCMC repeats and 10 replicate analyses for each value of K. The *viatica*17-east and P24(XY)-east pairing was analysed further with K = 1–4, 100,000 burn in runs, 1,000,000 MCMC repeats and 10 replicate analyses for each value of K in order to identify hybrid individuals reliably. We designated individuals as hybrids if their genotype was assigned as being <98% from their nominal parental population. This resulted in eight sample sites out of 20 in total for *viatica*17-east and P24(XY)-east assigned as exclusively containing hybrid individuals. One further individual from an otherwise exclusively *viatica*17-east sample site was assigned as a hybrid. In total, this resulted in 48 P24(XY)-east individuals, 45 *viatica*17-east individuals, and 51 hybrid individuals. Investigative STRUCTURE runs were also carried out between all pairs of chromosomal race populations, and within individual populations, with K = 1–4, 100,000 burn in runs, 500,000 Markov Chain Monte Carlo (MCMC) repeats, and 3 replicate analyses for each value of K. In all instances we assumed admixture, and provided no prior population information to assist clustering.

STRUCTURE HARVESTER version 0.6.7 [53], which uses the method of Evanno *et al*. [54] to infer levels of population structure, was used to define the uppermost hierarchical level of population structure across the whole island. Evanno *et al*. [54] suggested using the change in log likelihood between runs with different values of K in order to detect the true number of clusters. We used CLUMPP [55] to align and average cluster assignment of individual genome over independent repeats for each value of K.

### Outlier analysis

We conducted pair-wise outlier analyses for all ten population pairs of *V. viatica* on KI. These included four inter-race population pairs that have contiguous distributions, and form potential contact zones; four inter-race population pairs that do not have contiguous distributions; and two intra-race population pairs that do not have contiguous distributions. Outlier analysis was conducted using BayeScan 2 [33]. BayeScan 2 aims to detect loci under selection by comparing allele frequencies between populations, assuming they follow a multinomial Dirichlet distribution, which takes into account complex demographic models with varying gene flow between loci and between populations with different effective population sizes. The posterior probabilities of two models are compared: one including selection via a locus-specific F_ST_ component to explain observed allele frequency differences, and a ‘neutral’ model with only population-specific F_ST_ parameters which are shared across all loci. If the model including a locus-specific F_ST_ component is necessary to describe the observed allele frequencies, then a departure from neutrality is assumed for that locus. BayeScan runs were implemented using a uniform distribution of F_IS_ between 0–1, prior odds for the neutral model of 1, and default values for all other parameters, including 100,000 iterations in total, 50,000 of which consisted of a burn-in period. Following Jeffrey's scale of evidence [56], we considered a log posterior odds (log PO) greater than one as strong evidence for selection. The posterior odds is the ratio of posterior probabilities of the selection and neutral models and also allows the control of the False Discovery Rate (FDR) – the proportion of false positives among loci classified as under selection (BayeScan 2 manual). We checked that all loci classified as significantly differentiated at log (PO) > 1 remained significant after applying an FDR P <0.05 using the method provided with BayeScan 2. Population samples from the hybrid zone between *viatica*17-east and P24(XY)-east that included individuals with hybrid genotypes were removed prior to analysis.

### Linkage Disequilibrium

Tests for linkage disequilibrium (LD) between pairs of AFLP loci were performed with an in-house R script using the method of Li *et al*. [55] which uses an expectation maximization (EM) algorithm to obtain the maximum-likelihood estimates of LD between dominant markers. This script is available on request from Raj Whitlock (r.whitlock@liverpool.ac.uk). Loci with a level of polymorphism <10% were removed prior to analysis, because the calculation of LD is sensitive to low allele frequencies.

In order to test for pervasive genetic associations between loci which are differentiated between *viatica*17-east and P24(XY)-east, we compared the level of LD between pairs of outlier loci and pairs of putatively neutral loci in population samples that included hybrids, plus one additional hybrid individual: First, we approximated allele frequencies in P24(XY)-east and *viatica*17-east populations (excluding hybrid individuals) for all loci using the method of Zhivotovsky [57], implemented in AFLPsurv [46]. Then, we visualised the distribution of LD between all locus pair combinations in hybrid individuals against the product of the differences in allele frequencies between the same pairs of loci across the hybrid zone between *viatica*17-east and P24(XY)-east: [(p–q)*(r–s)], where **p** is the frequency of the presence allele of locus 1 in the parent population (excluding hybrids) of *viatica*17-east; **q** is the frequency of the presence allele of locus 1 in P24(XY)-east; **r** is the frequency of the presence allele of locus 2 in *viatica*17-east; and **s** is the frequency of the presence allele of locus 2 in P24(XY)-east. This product is directly proportional to the product of the allele-frequency slopes, provided that cline widths do not vary among loci (cf. [20]). Because we expect that LD = σ^2^b_1_b_2_/r (where σ^2^ is the variance of parent-offspring distances, b_i_ is the gradient in allele frequency for locus *i* and r is the recombination rate; [39]), any group of loci with lower average recombination than randomly chosen loci will have higher LD than expected from the product of the slopes and so from the product of the differences in allele frequency.

We compared the distribution of LD between outlier locus pairs with the distribution of LD between putatively neutral locus pairs that had a comparable product of allele frequencies across the hybrid zone, using two separate two-sample t-tests because the distribution of LD vs [(p–q)*(r–s)] appeared asymmetric. The samples for one t-test comprised pairs of outlier loci that were both fixed for the presence allele in one population, as well as neutral loci with a similar product of allele frequencies range: [(p–q)*(r–s)] <−0.75, and the samples for the second t-test comprised pairs of outlier loci that were fixed for presence alleles in opposite populations as well as neutral loci with a similar product of allele frequencies range: [(p–q)*(r–s)] >0.75.

## Supporting Information

Table S1 **Outlier loci comparison.** Outlier loci from pair-wise comparisons which were significantly differentiated between population pairs at log(PO) > 1 and FDR <0.05. Outlier loci from p24n vs v17s were not significantly differentiated, but did form a distinct cluster in the Fst-Log(PO) plot produced by BayeScan 2– see fig. 4B.
(XLS)
